# An evaluation of Canada's Compassionate Care Benefit from a family caregiver's perspective at end of life

**DOI:** 10.1186/1472-684X-7-14

**Published:** 2008-08-28

**Authors:** Valorie A Crooks, Allison Williams

**Affiliations:** 1 Department of Geography, Simon Fraser University, RCB 6141, 8888 University Drive, Burnaby, British Columbia, V5A 1S6, Canada; 2 School of Geography & Earth Sciences, McMaster University, 1280 Main Street West, Hamilton, Ontario, L8S 4M1, Canada

## Abstract

**Background:**

The goal of Canada's Compassionate Care Benefit (CCB) is to enable family members and other loved ones who are employed to take a temporary *secured *leave to care for a terminally ill individual at end of life. Successful applicants of the CCB can receive up to 55% of their average insured earnings, up to a maximum of CDN$435 per week, over a six week period to provide care for a gravely ill family member at risk of death within a six month period, as evidenced by a medical certificate. The goal of this study is to evaluate the CCB from the perspective of family caregivers providing care to individuals at end of life. There are three specific research objectives. Meeting these objectives will address our study purpose which is to make policy-relevant recommendations informed by the needs of Canadian family caregivers and input from other key stakeholders who shape program uptake. Being the first study that will capture family caregivers' experiences and perceptions of the CCB and gather contextual data with front-line palliative care practitioners, employers, and human resources personnel, we will be in a unique position to provide policy solutions/recommendations that will address concerns raised by numerous individuals and organizations.

**Methods:**

We will achieve the research goal and objectives through employing utilization-focused evaluation as our methodology, in-depth interviews and focus groups as our techniques of data collection, and constant comparative as our technique of data analysis. Three respondent groups will participate: (1) family caregivers who are providing or who have provided end of life care via phone interview; (2) front-line palliative care practitioners via phone interview; and (3) human resources personnel and employers via focus group. Each of these three groups has a stake in the successful administration of the CCB. A watching brief of policy documents, grey literature, media reports, and other relevant items will also be managed throughout data collection.

**Discussion:**

We propose to conduct this study over a three year period beginning in October, 2006 and ending in October, 2009.

## Background

The goal of Canada's Compassionate Care Benefit (CCB) is to enable family members and other loved ones who are employed to take a temporary *secured *leave to care for a terminally ill individual at end of life. It came into effect in January of 2004 through changes to the Employment Insurance Act and Canadian Labour Code. Its establishment was based on recommendations from a recent health care commission [1] as well as years of advocacy from the palliative care and caregiving communities. The CCB is administered through the federal Employment Insurance (EI) program as a 'special benefit'.

Successful applicants of the CCB program can receive up to 55% of their average insured earnings, up to a maximum of CDN$435 per week, over a six week period to provide palliative/end-of-life (P/EoL) care for a family member or other loved one at risk of death within a six month period. In order to qualify, applicants must have worked a minimum of 600 insurable employment hours over the previous 52 week period. Applicants must also meet the designation of 'family member'^1 ^and have access to a medical certificate from the gravely ill or dying individual's doctor, indicating that death is imminent (i.e., within a six month period), in order to be successful.

The six weeks of income assistance afforded by the CCB can be taken at once, broken down into one week periods and spread out over six months, and/or be shared between family members. Successful applicants must first go through a two week unpaid waiting period before starting payments. Also, a successful applicant must determine on his or her own when to request that the payments begin, with the first payment to be made within 28 days of beginning the claim.

The *goal *of this study is to evaluate the CCB from the perspective of family caregivers providing care to a terminally ill individual at end of life. Based on a successful pilot evaluation undertaken in 2005 involving interviews with 25 family caregivers [2-4], this study will use Patton's [5] comprehensive utilization-focused approach to evaluation. To this end, there are three specific research objectives:

(1) to examine the usefulness of the CCB for family caregivers providing P/EoL care and determine those elements of the program that can be changed/refined to better support their needs;

(2) to explore front-line palliative care practitioners' perceptions of the CCB, including the barriers and facilitators to use, and how they determine whether or not to recommend the CCB to family caregivers on a case-by-case basis; and

(3) to investigate barriers and facilitators inherent in the organization of specific workplaces and within the labour market in general that shape uptake of the CCB from the perspective of employers and human resources personnel.

Meeting these objectives will address our study purpose, which is to make policy-relevant recommendations informed by the needs of Canadian family caregivers and input from other key stakeholders who shape program uptake.

### Context

The primary goal of P/EoL care is to improve the quality of life and quality of death for dying people and their families through the provision of excellent care. Confirmed by a growing body of research, family units are assuming the majority of costs and responsibilities associated with P/EoL caregiving in an increasingly rationalized Canadian health care system [6]. The responsibilities associated with P/EoL caregiving are often more considerable than what family members can manage, resulting in compromised emotional, mental, social, financial and physical well-being [6-9]. Although many family caregivers want to provide care for their loved ones at the P/EoL stage, work interference can result in significant stress and burden [10] and many are faced with no provisions for paid leave and a lack of job security when returning to work. Furthermore, MacBride-King [11] reports that 48% of Canadian family caregivers find it difficult to balance caregiving and workplace responsibilities and 42% experience a great deal of stress in trying to meet the dual demands. The burden placed on family members has been shown to be of concern to P/EoL patients. For example, those patients surveyed in the study of Singer et al. [12] identified relieving caregiver burden as one of five elements of quality P/EoL care. Cohen and Leis [13] have also identified the burden placed on family caregivers to be a primary determinant of the quality of life of patients receiving P/EoL care. At the same time, contemporary shifts in the provision of P/EoL care from institutional settings to those in the community are resulting in increased family caregiver burden [14-16] which may outpace caregivers' individual capacities to cope [9,17,18].

While family caregivers ignoring their physical and mental health in order to provide P/EoL care is common [19], financial and workplace obligations are more difficult to disregard. For example, in addition to needing to pay for existing personal financial responsibilities during the caregiving period, often by maintaining involvement in paid labour, family members providing P/EoL care contribute, on average, CDN$6000 in unpaid caregiving during the final four weeks of life [20]. Grunfeld et al. [10] note that family caregivers caring for terminally ill patients not only experience depression and anxiety but also adverse work impacts, such as missing scheduled shifts, and typically financial burden, such as the cost of purchasing prescription drugs out-of-pocket. Thus, gaining access to financial support is a particular need of family caregivers providing P/EoL care [21] as such support minimizes financial stressors.

Canada's response to the growing demand to provide job and income support to family caregivers providing P/EoL care has been to develop the CCB. The CCB is a health-related social program that falls under the purview of Human Resource & Skills Development Canada. The CCB program and the legislative changes that shape its administration have come about in an era of neo-liberally informed social policy creation in Canada. An important ideology that underscores the creation of social policies in such a political climate is that families and voluntary agencies, rather than local states, should bear the onus of responsibility for assisting persons in need [22,23]. Another element of policymaking in the current neo-liberal climate has been the focus on providing care in the community as opposed to in institutional settings [24]. A significant outcome of this has been an increased reliance on the voluntary sector and unpaid labour in meeting such care needs [25]. It is these types of changes in the role of the state in Canadian society that have informed the development and implementation of the CCB – a program that facilitates care being provided in community settings by family members and other loved ones.

According to the Health Council of Canada, the CCB is a P/EoL initiative of international excellence [26]. At the same time, there has been a great deal of national criticism focused on the CCB program. Picard's [27] opinion piece in the national newspaper *The Globe and Mail*, for example, surmised the following:

A social program that provides some modicum of financial relief is entirely appropriate, and much needed. But the current program is not passing muster. It is unduly bureaucratic, inflexible and heartless. In short, the compassionate care program is utterly lacking in compassion.

Critics of the program have focused on a number of issues as reflecting this 'lack of compassion'; these include: the two week waiting period for payments [27] and the labour market participation requirements that cannot be met by family caregivers who have taken time off from work to provide long-term care [28], The Canadian Federation of Independent Businesses [29] has also identified concern about the amendment of the Canada Labour Code, expressing that regulating such leaves may negatively affect small businesses in particular due to their smaller workforces. Other concerns have been raised about the design and implementation of the CCB. A recent review of the CCB by The Health Council of Canada [26] has noted that one of the CCB's most significant issues is its problematically low uptake. Further, the Canadian Women's Health Network [30] has pointed out the gender-based disadvantage inherent in the program in that women are more likely to be ineligible for CCB income support because they are more likely to be stay-at-home parents and part-time workers who do not meet the CCB's eligibility criteria. Being the first study that will capture family caregivers' experiences and perceptions of the CCB and gather contextual data with front-line palliative care practitioners, employers, and human resources personnel, we will be in a unique position to provide policy solutions/recommendations that will address these and other concerns.

## Methods/design

As we are looking to gather lived experiences from both family caregivers and stakeholders who inform caregivers' experiences of the CCB, we propose to conduct an inductive study [31] using a qualitative approach. The method and techniques of data collection and analysis that we propose here are directly informed by the research objectives stated earlier. More specifically, we will achieve this goal through employing: Patton's utilization-focused evaluation as our methodology, in-depth interviews and focus groups as our techniques of data collection, and constant comparative as our technique of data analysis.

The objective of utilization-focused evaluation is to inform program and policy improvement using research findings. According to Patton [[5] p.20]:

*Utilization-Focused Evaluation *begins with the premise that evaluations should be judged by their utility and actual use; therefore, evaluators should facilitate the evaluation process and design any evaluation with careful consideration of how everything that is done, *from beginning to end*, will affect use. Nor is use an abstraction. Use concerns how real people in the real world apply evaluation findings and experience the evaluation process. Therefore, the *focus *in utilization-focused evaluation is on *intended use by intended users*. (emphasis in original)

This method is appropriate for our study as its purpose is to create policy recommendations which will directly affect family caregivers based on input from family caregivers and those who inform their uptake of the CCB, this being a focus on 'intended use by intended users.' There are twelve broad tasks that shape the method of utilization-focused evaluation; they are to: (1) determine readiness for assessment; (2) assess the readiness of the evaluators; (3) recruit an evaluation taskforce (ETF); (4) consider the evaluation context; (5) identify intended users; (6) determine the evaluation focus; (7) design the evaluation techniques; (8) test data collection techniques; (9) collect data; (10) analyze data; (11) facilitate the use of the findings; and (12) assess the evaluation process. Having already created an ETF that has worked with the research team to interpret the findings of the pilot study [2-4] (i.e., interviews conducted with 25 family caregivers regarding the CCB) and inform the direction of this full evaluation, we have already completed steps one through eight and are ready to move ahead with completing a full evaluation of the CCB from the perspective of family caregivers.

A foundational principle of the utilization-focused approach to evaluation is to have the research inform program improvement, not only through collecting relevant data but by increasing the ETF's commitment to employ the data for program improvement. The ETF members, all of whom were engaged in the pilot study, will continue in this role. As in the pilot evaluation, members of the ETF will work with the investigators to finalize data collection instruments, identify and recruit participants, interpret findings, and identify venues for knowledge transfer and translation. The work of the ETF will primarily be done via regular teleconferences, although communication via e-mail will take place between these meetings. The principal responsibility of the ETF will be to ensure that the evaluation produces policy-relevant findings that will: (1) have implications for family caregivers and their use of the CCB, and (2) be of use to key members of the P/EoL care policy community.

Upon completion of the pilot evaluation it was determined by the investigators and ETF that data collection will need to take place with three specific respondent groups in this full evaluation: (1) family caregivers who are providing or who have provided P/EoL care; (2) front-line palliative care practitioners; and (3) human resources personnel and employers. Each of these three groups has a stake in the successful administration of the CCB. Further, it is our contention that collecting data with all of these groups is essential in order to gain the fullest contextual understanding of the barriers and facilitators that shape family caregivers' uptake of the CCB while best informing how to better meet their needs. Data collection with each of these groups will be undertaken in five provinces: British Columbia, Manitoba, Newfoundland, Ontario, and Quebec. These provinces were carefully selected by the team upon completion of the pilot evaluation to represent Canada's regional diversity.

### Respondent Group #1: Family caregivers who are providing or who have provided P/EoL care

In-depth interviews are known to yield rich, nuanced data [32]. Further, in-depth interviews conducted by phone are known to be cost effective and produce reliable data [33,34]. We propose to conduct in-depth phone interviews with three groups of family caregivers: (1) successful CCB applicants; (2) unsuccessful applicants; and (3) non-applicants (i.e., retired, self-employed, or unemployed). Upon completion of the pilot study it was determined that collecting data from each of these three groups was relevant to the overall goal of evaluating the CCB from the perspective of family caregivers. We plan on accessing family caregivers from a wide variety of populations, including those providing P/EoL care for loved ones with cancer, Alzheimer's disease, and end-stage cardiopulmonary disease, in order to capture the diversity of caregiving experiences.

We will conduct interviews with five family caregivers from each group in each of the five provinces resulting in a total of 75 interviews (25 in each category, 15 from each province). The interviews will address: (a) to what extent family caregivers are satisfied with the CCB; (b) perceived strengths of the CCB; (c) recommendations for improving the CCB; (d) family caregivers' experiences of employers' responses to taking the CCB leave; and (e) the logistical elements of applying for and/or receiving the CCB. Interview guides were tested and refined during the pilot evaluation. We will also administer a demographic questionnaire which was used and refined during the pilot evaluation to capture standard information about the caregiving experience and the personal characteristics of the caregiver and care recipient.

### Respondent Group #2: Front-line palliative care practitioners

The pilot evaluation revealed that 20 of the 25 family caregiver interviewees had some degree of awareness of the CCB prior to participating in the study [2]. They had first learned of the CCB from a variety of sources, primarily from the media. We also found that there is a significant difference between being aware of the CCB's existence and having a working knowledge of both how the CCB is administered and its eligibility requirements. Many participants lacked this kind of knowledge. An important group of professionals who have the capacity to share this type of information with potential applicants are front-line palliative care practitioners. For the purpose of this study, we define this group as including clinicians (e.g., nurses, nurse practitioners, family doctors, palliative care specialists), social workers, bereavement councillors, and P/EoL coordinators/program managers. Upon completion of the pilot evaluation it was determined that in the full evaluation, data collection would need to take place with these key informants as they provide important contextual data for the evaluation of the CCB from the perspective of family caregivers. We also believe it is important to consult with these key informants around barriers and facilitators of program uptake as they were some of the strongest advocates for developing the CCB and getting its supporting legislation enacted; thus, they have a useful perspective to contribute.

We propose to conduct phone interviews with 50 key informants, ten in each of the five provinces. The interviews will address the following: (a) perceptions of the CCB's usefulness and barriers/facilitators to access; (b) experiences of recommending the CCB to a client/client's family; (c) working knowledge of the CCB's administration and eligibility requirements; and (d) suggestions for improvement. The investigators will work together with members of the ETF in the first year of the project to develop an instrument for data collection that addresses these and related issues. The instrument will also be informed by preliminary findings with the family caregiver respondent group.

### Respondent Group #3: Human resources personnel and employers

Focus groups are known to have many benefits [35,36], including that participants are given the opportunity to engage in discussion with others about a topic of mutual interest. We propose to conduct five focus groups with human resources personnel and employers, one in each of the five study provinces. For the purpose of this study, we consider human resources personnel to be those individuals who take care of payroll, labour management, and/or administering benefits within the company with which they are employed or who work for a human resources management firm. We consider employers to be those individuals who have the ultimate responsibility for managing employees, including hiring/firing and negotiating leaves, within a company they own, direct, and/or manage. Upon completion of the pilot evaluation, the research team and ETF determined that this was another important stakeholder group to target. More specifically, this group will provide us with important contextual information about the logistics of having an employee take leave through the CCB, as well as offering insights into the CCB's usefulness from a labour market perspective.

The focus groups will be run with 7–10 participants in each. Thus, we expect to collect data with anywhere from 35 (7 respondents/focus group) to 50 (10 respondents/focus group) employers and human resources personnel. Topics to be covered in the focus groups include: (a) perceptions of the CCB's usefulness and barriers/facilitators to access; (b) experience with having an employee take the CCB; (c) working knowledge of the CCB's administration and eligibility requirements; (d) strategies for supporting employees who are providing P/EoL care while involved in paid labour; and (e) suggestions for improvement. The research team will work together with members of the ETF in the first year of the project to develop a focus group guide that addresses these topics. The instrument will also be informed by preliminary findings with the family caregiver respondent group.

### Watching brief

A watching brief of policy documents, grey literature, media reports, and other relevant items will be managed throughout the period of data collection. These sources will be accessed through conducting frequent searches for updated sources in media and publication search engines. Furthermore, members of the ETF will contribute relevant documents such as newsletters and policy briefings generated by their respective offices and organizations. The purpose of conducting the watching brief is to keep up-to-date on issues of relevance to the CCB including legal appeals and policy changes. These secondary data will assist in tracking any changes to the CCB and, in so doing, shape the policy context and augment our analyses of the three primary datasets.

### Recruitment: Telephone interviews

We will identify family caregiver participants in each of the three categories through recruitment strategies that were shown to have success in the pilot evaluation by engaging in both purposeful and snowball sampling. Our first step will be to disseminate calls for participants using the collective resources of the research team, ETF, and the Canadian Institutes of Health Research funded New Emerging Team in Family Caregiving for People at End of Life (NET). The targeted group will be individual caregivers who meet sampling criteria and to those service providers and organizations that have contact with our population of interest. In addition to recruiting through the NET website via a posted advertisement, strategic internet searches will also be undertaken to identify community-based organizations in each of the five provinces that provide services for our target population (e.g., local support groups, family caregiver networks). French and English advertisements will be circulated to these groups via e-mail. We will then place advertisements in provincial newspapers. We will also snowball out from other participants by asking them if they know of anyone else who might be interested in participating in the study. Our last tested strategy will be to send letters to the offices of Members of Parliament in the target provinces to make them aware of the study and ask them to post a recruitment advertisement in their local offices and to share information about the project with any constituents they know who meet our sampling criteria. We found this to be useful in the pilot project as it assisted in identifying those who had applied for the CCB, as some had shared their experiences and even complaints with the constituency office.

We will identify members of the key informant group (i.e., front-line palliative care practitioners) using the extensive networks which exist in the research team, ETF, and the NET. As with the family caregiver group, we will circulate advertisements in French and English through our collective networks and to newsletters and listservs of relevant organizations. We will also contact directly key informants with whom there is already a working relationship established.

### Recruitment: Focus groups

The identification of employers and human resources personnel to participate in the focus groups will be done through established linkages with relevant professional associations. Advertisements about the focus groups will be circulated in French and English through these associations. To minimize logistical arrangements in meeting with active members, we aim to conduct the five focus groups at the provincial human resources association meetings, likely before or after conference sessions on a day agreed upon by all participants. We will rent a room at the conference location in which to host the focus group. We will run four English-language focus groups and one French-language focus group.

### Informed Consent

Because the interviews with family caregivers and front-line palliative care practitioners will take place by phone, verbal consent will be sought. After scheduling an interview, these participants will be mailed or e-mailed a detailed information letter that contains information on their rights as participants. At the start of the phone interview the interviewer will review these details and read a consent script in order to obtain verbal consent. At this point the interviewer will sign a consent form indicating that the script was read and verbal consent has been granted. The respondent will have received a copy of this form in the letter of information package to keep for his/her own records. Human resource personnel will similarly be mailed or e-mailed a detailed letter of information with details of the study, participant rights, and the focus group information. Because these groups will happen face-to-face, a signed consent form will be used. A confidentiality script will be read aloud at the start of the group reminding participants that discussion that happens in the group is to remain confidential. These procedures have been reviewed and approved by the Office of Research Ethics at Simon Fraser University (certificate #37980) and Research Ethics Board at McMaster University (certificate #2006172).

### Data Management & Analysis

All interviews and focus groups will be audio taped. Data analysis will proceed with the verbatim transcription of all interviews and focus groups which will be imported into the qualitative data management program NVivo. NVivo has been selected for data management because it allows for collaboration between researchers at multiple sites as in the case of our research team. All researchers and trainees involved in the project will have some form of involvement in data analysis, whether to analyze the findings of a particular respondent group or to collaborate on a particular element of the analysis (e.g. interview themes/codes) to ensure investigator triangulation. Investigator triangulation of this nature will also assist us in enhancing the reliability of the findings [37].

Analysis of all focus groups and interviews with participants and key informants will be guided by the constant comparative technique. While this technique was originally developed to be used with the grounded theory method as a way to engage in analysis while data collection is ongoing [38,39], it has usefully been adopted in other types of qualitative approaches [39]. In our study we will use this technique to analyze completed datasets (i.e., data collection and analysis will not be concurrent). It is an appropriate technique as it provides a way to move beyond description of qualitative data and toward explanation, in that comparing findings between groups and explaining differences will allow us to shape the most relevant policy recommendations. This analytic technique "involves taking one piece of data (one interview, one statement, one theme) and comparing it with all others that may be similar or different in order to develop conceptualisations of the possible relations between various pieces of data" (p. 69) [39]. Our constant comparative analysis will take place at three levels: (1) the intra-group, (2) the inter-group, and (3) the inter-topic. Undertaking multiple types of comparisons within the same project is a common component of this analytic technique [38].

## Discussion

We propose to conduct this study over a three year period beginning in October, 2006 and ending in October, 2009. During the first year (October 2006–07) we will undertake data collection with family caregivers (n = 75) while working to identify potential participants from the other groups (key informants, human resource personnel, and employers). Upon completing collection of these data, we will undertake analysis of the dataset. In the first year (October 2006–07) we will also develop the instrument for collection of data with the key informants and the probes for the focus groups with human resources personnel and employers, both of which will be informed by the preliminary findings of the family caregiver dataset. From October, 2007 through to February, 2008 we will conduct data collection with the key informants, namely front-line palliative care practitioners (n = 50). Full analyses of these data will begin once all interviews have been conducted. We expect to collect focus group data from human resource personnel and employers during the second year (October 2007–08) by attending provincial association conferences that take place during this period (n = 5 focus groups). All data collection will be completed by October 2008 and all analysis will be completed by March 2009.

In the final year of the study (October 2008–09), we will work collectively to interpret the data from which policy directions arise. Appropriate venues for dissemination will also be identified. We will also work at this time to assess the evaluation process which is, as described in the study details section, an important element of Patton's [5] utilization-focused evaluation, during the final year. Throughout the three years the watching brief will be updated regularly and will be used to inform the analysis and identification of significant findings. In addition to widely disseminating a full and summary report during the final year, findings will be presented at scholarly and policy conferences and manuscripts will be submitted to peer-reviewed national and international journals. Final research reports and summaries will be made available in both English and French and posted on the NET website . Further, members of the ETF will assist with disseminating research products, distributing them to their membership and other key stakeholders. The Canadian Hospice Palliative Care Association, of which all provincial palliative care associations are members, will play a particularly central role in this regard, advocating for changes to federal government officials in Ottawa. Numerous other organizations have been identified for report dissemination.

## Abbreviations

P/EoL: palliative/end-of-life; ETF: evaluation taskforce; NET: New Emerging Team in Family Caregiving for People at End of Life; CCB: Compassionate Care Benefit.

## Competing interests

The authors declare that they have no competing interests.

## Authors' contributions

VC and AW contributed equally to the design and writing of this protocol.

## Appendix 1

### Endnotes

^1 ^At the time this research protocol was written and submitted for funding there was a limited definition of 'family member' that included only immediate relatives (e.g., parents and children) and not siblings, aunts, uncles, cousins, and other relatives. Since funding was obtained the range of eligible caregivers was broadened extensively to include not only all family members, including foster parents and in-laws, but also any loved one deemed as family by the dying individual being cared for. We retain our use of the term 'family caregiver' here because all caregiver respondents for that part of the study were indeed members of either the nuclear or extended family of the dying individual.

## Pre-publication history

The pre-publication history for this paper can be accessed here:



## Supplementary Material

Additional file 1 Peer review reports. This file contains the external review reports collated by CIHR for this protocol. Click here for file
