# Supplementary material for: An evaluation of Canada's Compassionate Care Benefit from a family caregiver's perspective at end of life
Source: BMC Palliat Care. 2008 Aug 28;7:14. doi: 10.1186/1472-684X-7-14 (PMC2533295; doi:10.1186/1472-684X-7-14)
Supplement: Additional file 1 — Peer review reports. This file contains the external review reports collated by CIHR for this protocol. [file 1472-684X-7-14-S1.pdf]

Review Type/Type d'évaluation: SO Notes /Notes de l'agent scientifique
Name of Applicant/Nom du chercheur: WILLIAMS, Allison Marie
Application No./Numéro de demande: 159760
Agency/Agence: CIHR/IRSC
Competition/Concours: 2006-03-01 Operating Grants/Subventions de fonctionnement
Committee/Comité: Palliative and End of Life Care/Les soins palliatifs et les soins de fin de vie
Title/Titre: An evaluation of Canada's compassionate care benefit from a family caregiver's perspective

---

Assessment/Évaluation:

The Compensation Care Benefits (CCB) program is the subject of this research project. Ethnographic qualitative analysis will be used. A prior pilot study was conducted, including applicants (successful & unsuccessful) and non-applicants; this allowed highlighting of some of the problems and barriers to participation in the CCB program. A new and broadened sample will be included in the current study. Participants in 5 Canadian provinces will be recruited. Application is well written, purpose is clearly stated, timely and likely to produce results that are socially important.

Up to date and internationally recognized program evaluation approaches will be used. Findings may help shape social policy. Research will complement other research activities of the Family Caregiver NET.

Methods are relevant. The involvement of trainees, the development of a new cadre of research capacity in this area, was seen as an asset.

Suggest an external advisory board for the project to interpret the findings with respect to policy, to remove any perception of potential bias. This would lend credibility to the conclusions and recommendations.

The addition of quantitative data may be of interest, such as Likert scales during interviews to provide a scale of the importance of issues raised by the family.

Review Type/Type d'évaluation: Committee Member 1 /Membre de comité 1
Name of Applicant/Nom du chercheur: WILLIAMS, Allison Marie
Application No./Numéro de demande: 159760
Agency/Agence: CIHR/IRSC
Competition/Concours: 2006-03-01 Operating Grants/Subventions de fonctionnement
Committee/Comité: Palliative and End of Life Care/Les soins palliatifs et les soins de fin de vie
Title/Titre: An evaluation of Canada's compassionate care benefit from a family caregiver's perspective

---

Assessment/Évaluation:

A.Assessment of the applicant
The two PI's on this proposed research are Dr. Allison Williams and Dr. Valorie Crooks. Dr. Williams is an Associate Professor in the Department of Geography and Geology at McMaster University. She is a CIHR New Investigator with expertise in health systems/services research and maintaining family caregiver health in palliative and EOL care. Dr, Williams has several primary authored peer reviewed papers and she has presented extensively on topics that are commensurate with her research interests. She is currently Co-applicant on a CIHR NET on family caregiving in palliative care and EOL (Stajduhar) and a PI on a CIHR ICE grant re timely access and transitions in rural POL care. Dr. Allison is also PI or Co-applicant on other tricouncil grants re survey instrument development, quality of life and social policy. Dr. Crooks is an Assistant Professor in Geography at Simon Fraser University. She is a New Investigator on the aforementioned CIHR NET and Co-applicant on the CIHR ICE grants. Her areas of expertise include social policy and qualitative methods. Dr. Crooks has 3 peer reviewed papers published (one as primary author) and one in press.

The other four applicants on this 3 year multi-site proposal are all skilled researchers with backgrounds in family caregiving, evaluation research and qualitative and focus group methodology. Study sites include BC, Manitoba, Newfoundland, Ontario and Quebec.Drs. Williams and Crook will manage data collection and have each committed 10hr/wk to this project. All co-applicants will assist with recruitment. Drs. Stajduhar, Allan, and Cohen will assist with focus group and analysis of qualitative data. Dr. Cohen is fluent in French and will manage recruitment and focus group in Quebec. Dr. Brazil, who is a seasoned expert in evaluation research, will oversee the research process providing support as needed.

B.Synopsis of the proposal
Background/Purpose
Caregiver burden associated with palliative and EOL impacts the health of the caregiver and it renders them financially vulnerable. In an attempt to alleviate some of the financial burden, the Canada Compassionate Care Benefits (CCB) was established in 2004 as a "special benefits" administered through the federal unemployment insurance program. The CCB allows provincial and territory administration to support 6 weeks employment insurance benefits and job security to family caregivers who take time off work to care for a family member who is at risk for dying within a six month period.

In 2005, only 4% of the annual CCB budget was claimed despite increased reliance on communities and individuals to provide palliative and EOL care to affected individuals. The low uptake is thought to be due to provincial labor legislation and consumer access and eligibility. To date, 3 of the 13 Canadian provinces have not amended their job protection legislation and the current definition of what constitutes a "family member" is exclusionary. Also excluded are family caregivers who are retired, self-employed or unemployed. Successful application however does not guarantee timely or sustained CCB. Applicants must obtain a medical certificate indicating that death is imminent (within 6 months), they must go through a 2 week unpaid period and they must decide on their own when first payment is to begin.

The purpose of this proposed study is to make policy relevant recommendations to the CCB based on the self reports of the intended family caregiver users of CCB and other key stakeholders who shape CCB uptake. It is based on a prior pilot that targeted family caregivers caring for non-cancerous and chronically ill patients and that employed Patton's utilization focused approach to evaluation. Specific research objectives are to:

1.examine the usefulness of the CCB from the family caregiver's perspective with a view to determine which elements can be changed to better support their needs
2.explore front-line POL practitioner perspective of the CCB to determine whether or not to recommend CCB to family caregiver's on a case-by-case basis
3.explore the perspectives of employers and human resources personnel with a view to investigating barriers and facilitators inherent in the workplace and labor market that shape the uptake of CCB

Approach
Qualitative - ethnographic approach (purposive saturation sampling with constant comparative analysis of semi-structured telephone interview and focus group data.

Progress to date:
In 2004, the applicants conducted a pilot study using 8 of the 12 steps of the utilization-focused evaluation and constant comparative approaches to evaluate the CCB from the perspectives of three categories of family caregivers residing across Canada including 16 non-applicants, 4 successful applicants and 5 participants who applied to CCB but were unsuccessful (total n=24). Findings revealed several challenges faced by study participants including difficulty accessing reliable and accurate information, not knowing how to complete the CCB forms and having to go through the application process while contending with stressors associated with caregiving. Other findings related to the exclusionary family criteria, inadequate period of coverage and difficulty in obtaining a medical certificate of imminent death particularly if the ill person suffered from a prognostically challenging disease such as Alzheimer or congestive heart failure.

The current study will further the pilot evaluation by collecting data on a new and broadened sample of key-stakeholders, analyzing the data, facilitating use of the findings and assessing the evaluation process. The applicants plan to compare family caregiver perception before and after the anticipated broadening of the definition of "family caregiver". They propose sampling the same three categories of family caregivers - this time including those caring for persons with cancer. They also plan to sample additional key stakeholders such as practitioners and human resource personnel to help identify contextual and process issues re CCB uptake and areas for policy solutions and recommendations. The study samples will be collected from respondents residing in 5 provinces including those that have not legislated job security yet for family caregivers. This will enable the team to consider variations in access to social/health programs, supports and services within the same national administration. The team also plans to continue employing the utilization focused evaluation approach facilitated by an existing taskforce as they believe this will enable them to engage directly making use of findings to inform and improve CCB policy recommendations which will directly affect family caregivers.

C.Assessment of the proposal
Significance
This study employs up-to-date and internationally recognized program evaluation frameworks and philosophies. Conduction of this research would complement other activities related to the CIHR NET grant on family caregiving and has the potential to contribute importantly to the emerging body of knowledge on palliative and EOL care in Canada specific to national support programs. In specific, findings may help shape social policy and the administration of the CCB which is not fully or optimally utilized by its intended end users.

Originality: It appears that this group is one of the first to seek family caregiver perspectives to evaluate the CCB using a utilization evaluation approach. The strength of this study lays in novel use of a utilization approach to evaluation by a group knowledgeable about the current science on palliative and EOL caregiver burden and who have expertise in evaluation and social policy

Research Plan
The investigators do a good job of detailing and explaining who and how they will recruit, they explain approaches to qualitative analysis and the steps they will take to optimize uptake of the evaluation to improve CCB and its utilization. Their description of sample and sampling framework is feasible and they provide a reference for the appropriate use of constant comparative approach to the analysis of the three levels of qualitative data (between groups, within groups and between topics). In their plan, the applicants propose to draw on the panel of evaluation experts who were involved in the original pilot. The rationale for inclusion of this task force and its function is well explained, it is consistent with evaluation framework proposed and it will optimize uptake and application. This reviewer, however, advises inclusion of an assessment board external

to the study as this may add credibility to the overall study and policy recommendations. To this reviewer, the assessment component (step #12), in general, is not well explained. It is noted that the proposed study relies almost exclusively on the generation of qualitative data. While these forms of data are appropriate and required for explanatory purposes, the addition of quantitative measures could help strengthen the qualitative findings while providing opportunity to further comparative study analyses (the use of multi-methods is recommended by Patton). It may be that the applicants intend to generate non-qualitative data from the varying kinds of information they will collect (outside that of the demographics), but this was not made sufficiently clear. The applicants also aim to recruit English and French speaking key stake-holders. In doing this however they will exclude non-English and French speaking family caregivers who may be particularly vulnerable to access and uptake inequity of the CCB. The applicants will need to address this sampling issue perhaps via subsequent study.

Research Environment
The research environments have been strategically chosen and the researchers have established contacts on the basis of the pilot and their other work to enable successful completion of the study as proposed. The team is composed of a strong group of experts who have the knowledge, skills, resources and the support they need to complete this 3 year multi-site study.

In sum, this is an important, innovative and relevant research based on prior work and being carried out by an expert and committed team. It is well written. It offers to strengthen preliminary evaluation of a national program through the eyes of key stake holders and involve policy decision makers that would benefit the intended use of the program by its intended users.

Budget: The budget is well explained and detailed. While research training is to be commended, reliance on graduate and undergraduate summer students to assist with recruitment, coordination and other study related procedures may be risky (no allowance made for research assistants).

Review Type/Type d'évaluation: Committee Member 2 /Membre de comité 2
Name of Applicant/Nom du chercheur: WILLIAMS, Allison Marie
Application No./Numéro de demande: 159760
Agency/Agence: CIHR/IRSC
Competition/Concours: 2006-03-01 Operating Grants/Subventions de fonctionnement
Committee/Comité: Palliative and End of Life Care/Les soins palliatifs et les soins de fin de vie
Title/Titre: An evaluation of Canada's compassionate care benefit from a family caregiver's perspective

---

Assessment/Évaluation:

A: Applicant: Well funded, successful researcher at near end of first decade of post doctoral career and now an established researcher recently appointed at McMaster University.

B: SYNOPSIS

There is a clear focus on the overall purpose of the study through its stated and achievable goals. This study is about the value of the compassionate care benefit from perspectives of family caregivers. Powerful team of established researchers who make a strong case for their proposal within the mandate of the CIHR ..."to assist in the creation of new knowledge and its translation into improved health for Canadians." Policy recommendations can be expected.

Purpose:

To evaluate the Compassionate Care Benefit program though utilization-focused evaluation. The study will be conducted in 5 provinces with successful, unsuccessful and non-applicants to CCB program. Strengths and weaknesses of the program will be explored with caregivers of patients with diverse EOL needs/diagnoses plus focus group discussions with human resources personnel (identified as key stakeholders in the pilot work) and palliative care practitioners. Semi-structured interviews.

Context is clearly described with balanced overview of the program and challenges faced.

This team has already completed some vital pilot work through their NET using an approach they want to extend in the current study. They will be working with a powerful team of bureaucrats from Health Canada and other agencies. These agencies will guide them through a process of collating relevant policy and other documents during the study period.

Inclusion criteria

Choice of provinces is well justified. There will be 75 interviews in 5 provinces of three groups of applicants/non-applicants. The interview process, the use of focus groups, are all well justified. The recruitment strategy is impressive in its diversity and depth.

C: ASSESSMENT OF PROPOSAL

Overall comment: This proposal was a pleasure to read.

Significance: The applicants make a good case for the timeliness of this proposal as amendments to the CCB are in the works, mostly in regard to who is defined as the family member. Moreover they state but do not overstate the case for their ability to make recommendations for improvement to this important program.

Originality: Quite original in its scope

Analysis: Again the proposed approach is well described and the constant comparative technique seems appropriate to the subject matter.

Overall there is a clear description of the proposed analytic approach to qualitative data.

Timelines: Logical and should be met by this team

Research environment:

This is a team with great potential to make significant contribution to the field of policy evaluation. Their completion of necessary pilot work underscores the likely success of this project across 5 provinces. Many team members are accomplished researchers in their own right and as a team they will supervise two junior trainees at McMaster and Simon Fraser Universities.

Budget:
Much of the budget relates to support of student trainees and this is laudable. It seems reasonable to me

**Review Type/Type d'évaluation:**	SO Notes /Notes de l'agent scientifique
**Name of Applicant/Nom du chercheur:**	WILLIAMS, Allison Marie
**Application No./Numéro de demande:**	159760
**Agency/Agence:**	CIHR/IRSC
**Competition/Concours:**	2006-03-01 Operating Grants/Subventions de fonctionnement
**Committee/Comité:**	Palliative and End of Life Care/Les soins palliatifs et les soins de fin de vie
**Title/Titre:**	An evaluation of Canada's compassionate care benefit from a family caregiver's perspective

---

Assessment/Évaluation:

The Compensation Care Benefits (CCB) program is the subject of this research project. Ethnographic qualitative analysis will be used. A prior pilot study was conducted, including applicants (successful & unsuccessful) and non-applicants; this allowed highlighting of some of the problems and barriers to participation in the CCB program. A new and broadened sample will be included in the current study. Participants in 5 Canadian provinces will be recruited. Application is well written, purpose is clearly stated, timely and likely to produce results that are socially important.

Up to date and internationally recognized program evaluation approaches will be used. Findings may help shape social policy. Research will complement other research activities of the Family Caregiver NET.

Methods are relevant. The involvement of trainees, the development of a new cadre of research capacity in this area, was seen as an asset.

Suggest an external advisory board for the project to interpret the findings with respect to policy, to remove any perception of potential bias. This would lend credibility to the conclusions and recommendations.

The addition of quantitative data may be of interest, such as Likert scales during interviews to provide a scale of the importance of issues raised by the family.
